# Complete Genome Sequence of *Pontibacter akesuensis* Strain AKS 1^T^, Which Exhibits Robust Nutrient Metabolism in Harsh Environments

**DOI:** 10.1128/genomeA.00997-16

**Published:** 2016-10-06

**Authors:** Yang Wang, Kaiyong He, Yongzhong Jiang, Jiate Shen, Bo Yu

**Affiliations:** a Hubei Provincial Center for Disease Control and Prevention, Wuhan, China; b Hubei Institute for Food and Drug Control, Wuhan, China

## Abstract

*Pontibacter akesuensis* strain AKS 1^T^ was found in Akesu, Xinjiang Province, China, and exhibits the extraordinary ability to metabolize various substrates and is resistant to solar radiation. To gain insight into the bacterial genetic determinants for this adaptability, we report the complete genome sequence of strain AKS 1^T^.

## GENOME ANNOUNCEMENT

Akesu is a city in the Xinjiang Province in northwest China, and the microorganisms in that place exhibit extraordinary resistance to low humidity, high temperature variation, solar radiation, and poor nutrients. *Pontibacter akesuensis* strain AKS 1^T^ (available from the China Center for Type Culture Collection, CCTCCAB 206086^T^) was selectively isolated from the desert soil of Akesu by using a traditional dilution plating method. After Biolog GN2 tests, AKS 1^T^ indicated outstanding carbon utilization ability on 70 different compounds (1). In order to understand the genes related with nutrient metabolism, whole-genome sequencing was performed.

The genomic DNA of *P. akesuensis* strain AKS 1^T^ was sequenced by an Illumina Hiseq2000, and whole-genome shotgun (WGS) sequence libraries were prepared for two types with 100 bp and 500 bp insert sizes. WGS sequence data of 780 Mb, giving approximately 167-fold genome coverage, was generated and assembled into 32 contigs, which were connected into two scaffolds based on the paired-end relationships of libraries by using SOAPdenovo (2). Then, the structure of chromosome and plasmid was constructed and the gaps were filled by PCR-amplification. After finishing the complete genome of AKS 1^T^, the protein-coding sequences were predicted by Glimmer3.0 (3) and the function of genes were determined by BLASTp analysis with nr (4), gene ontology (GO) (5), KEGG (6), clusters of orthologous groups (COG) (7), and Swiss-Port (8) databases. In addition, the sequences of rRNA, tRNA, and tandem repeats were also predicted by RNAmmer (9), tRNAscan (10), and Tandem Repeats Finder (11) respectively.

The complete genome of *P. akesuensis* strain AKS 1^T^contained one circular chromosome (4,713,421 bp with a G+C content of 51%) and one plasmid (116,138 bp with a G+C content of 51.68%). The chromosome of AKS 1^T^contained 4,240 predicted genes, 12 rRNA operons, and 43 tRNAs. A total of 146 genes, zero rRNA operons, and zero tRNA were identified in the plasmid. Besides 14 microsatellites, 126 tandem repeats with a total length of 6,647 bp were predicted in the chromosome of AKS 1^T^.

From the gene annotation results of *P. akesuensis* strain AKS 1^T^, 2,375 genes were identified by the KEGG database. Meanwhile, 394 genes, 376 genes, and 233 genes, which were clustered using the functions “carbohydrate metabolism,” “amino acid metabolism,” and “replication and repair” separately, were the three main classification items. Furthermore, the strain contained 206 genes and 194 genes, which could be classified as “xenobiotics biodegradation and metabolism” and “energy metabolism.” Exploring the genes related to compound utilization in cruel environments, we found many genes belonging to the carbon-nitrogen hydrolase family or carbonic anhydrase family. Additionally, gene *aspB*, which functions as a carbon fixer in photosynthetic organisms, was also found in AKS 1^T^. These data provided a comprehensive description of the outstanding ability of nutrition metabolism and explained the adaption ability of AKS 1^T^ in harsh environments.

### Accession number(s).

Genome information for the chromosome and plasmid of *P. akesuensis* strain AKS 1^T^ was deposited in GenBank under the accession numbers CP014766 and CP014767, respectively.
